# Beyond diabetes and obesity: GLP‐1 receptor agonists in disrupting the vicious cycle of metabolic dysfunction and neuroinflammation

**DOI:** 10.1111/dom.70400

**Published:** 2025-12-19

**Authors:** Renata Spezani, Carlos A. Mandarim‐de‐Lacerda

**Affiliations:** ^1^ Laboratory of Morphometry, Metabolism, and Cardiovascular Disease Institute of Biology, Biomedical Center, The University of the State of Rio de Janeiro Rio de Janeiro Rio de Janeiro Brazil

**Keywords:** insulin resistance, metabolic dysfunction, microglia/glial modulation, neurodegenerative diseases, synaptic resilience

## Abstract

Neurodegenerative diseases, including debilitating conditions like Alzheimer's and Parkinson's, are characterized by progressive neuronal loss, a process fundamentally driven by persistent chronic neuroinflammation and central metabolic dysfunction. In these disorders, persistent danger signals, such as the aggregation of misfolded proteins, activate resident microglial cells, leading to a functional shift toward a detrimental, pro‐inflammatory phenotype. This damaging cycle is critically exacerbated by impaired Insulin/Insulin‐like Growth Factor 1 signalling, which compromises neuronal mitochondrial homeostasis, decreases energy production, and severely diminishes synaptic plasticity, thereby establishing a self‐perpetuating cycle of metabolic disturbance and neuroinflammation. This review examines the burgeoning therapeutic potential of Glucagon‐Like Peptide‐1 Receptor Agonists (GLP‐1RAs), a class of drugs traditionally used to manage type 2 diabetes mellitus and obesity, as neuroprotective agents. We discuss mechanistic insights demonstrating how GLP‐1RAs operate through a crucial dual action: effectively mitigating central insulin resistance and directly suppressing the multi‐faceted neuroinflammatory cascade. By activating specific neuronal and glial signalling pathways, GLP‐1RAs are shown to restore mitochondrial function, increase neuronal resilience, and crucially, modulate adverse glial cell responses—inhibiting the release of major pro‐inflammatory cytokines and significantly reducing cellular oxidative stress within the central nervous system. Clinical trials and comprehensive preclinical data, analysed through diverse experimental models of neurodegeneration, strongly support the translational potential relevance of these compounds. The accumulating evidence suggests that GLP‐1RAs offer a promising, readily available therapeutic strategy to disrupt the core inflammatory and metabolic pathways common across many neurodegenerative conditions, warranting further investigation in large‐scale human trials.

## 1 INTRODUCTION

Neuroinflammation refers to the complex immune response in the central nervous system (CNS) triggered by diverse challenges that activate the brain's innate defence mechanisms.^1^ Among the primary triggers are misfolded or aggregated proteins, which tend to accumulate in the context of several neurodegenerative disorders and act as persistent danger signals.^2^

A central component of this response is mediated by microglia, the resident macrophage‐like cells of the CNS. Under physiological conditions, microglia continuously survey the neural environment, contributing to synaptic remodelling, debris clearance, and maintenance of tissue homeostasis.^3^ However, upon sensing inflammatory stimuli, these cells undergo profound changes in phenotype and function, shifting toward a reactive state that orchestrates the production of cytokines, chemokines, and other mediators.^4^ Through these activities, microglia serve as both guardians of CNS integrity and principal drivers of neuroinflammation, linking innate immune activation to the progression of neurological disease.^5^

The hippocampus plays a central role in memory and emotion, and a distinctive feature of this region is its ability to generate new neurons throughout life within the dentate gyrus.^6^ These neurons progressively mature and incorporate into preexisting networks, enhancing the plasticity of hippocampal circuits.^7^ In concert with the ventromedial prefrontal cortex, the hippocampus also integrates emotional and reward‐related information into experiences, thereby supporting the transformation of episodic memories into semantic and autobiographical forms.^8^

The relationship between type 2 diabetes mellitus (T2DM), insulin resistance, and neurodegenerative processes has generated growing interest in therapeutic strategies that address both metabolic dysfunction and neuronal vulnerability.^5^ Incretin‐based therapies, initially developed to improve glycemic control, have shown additional properties, including reduced neuroinflammation, enhanced synaptic plasticity, and protection against protein aggregation.^9^ These actions provide a compelling rationale for exploring incretin analogs not only as metabolic regulators but also as potential neuroprotective agents, positioning them as promising candidates for disease‐modifying interventions in Alzheimer's disease and related disorders.

## 2 NEUROINFLAMMATORY MECHANISMS IN NEURODEGENERATION

Although the hippocampus is one of the earliest and most vulnerable structures affected in insulin‐resistant neurodegeneration, the cellular mechanisms described in this section—including microglial activation, astrocytic reactivity, mitochondrial dysfunction, and synaptic degeneration—are not restricted to this region. These processes occur throughout multiple cortical and subcortical areas and represent core, brain‐wide pathological drivers. The hippocampus is therefore presented as a representative, metabolically vulnerable model region within a broader CNS‐wide pathogenic landscape, while the mechanistic discussions apply to broader neuroinflammatory circuits.

### 2.1 Microglial activation and inflammatory pathways

Microglia originate from yolk sac‐derived myeloid progenitors during embryonic development and populate the brain parenchyma early in ontogenesis.^10^ In mice, microglia arise from a population of yolk sac–derived macrophages generated during primitive haematopoiesis, which begin migrating into the neuroepithelium at embryonic day 8.5. In humans, microglial progenitors enter the developing brain between 4.5 and 5.5 weeks of gestation.^11^ Under physiological conditions, microglia display fine, highly ramified processes, a state commonly referred to as “resting microglia”. In this form, they establish direct contact with neuronal synapses, continuously monitoring and adjusting to neuronal activity.^12^

**FIGURE 1:**
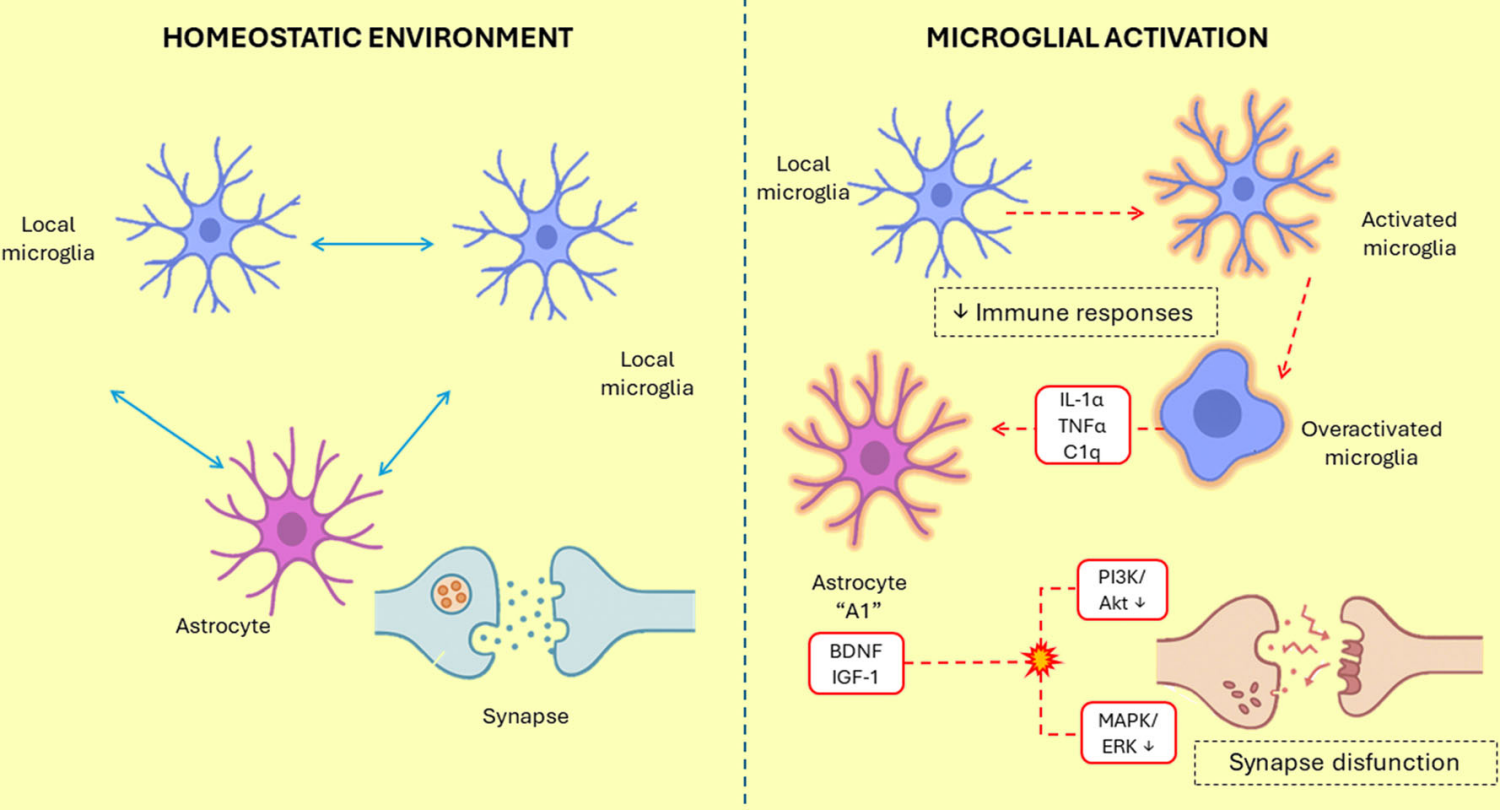
Microglial activation and astrocytic response in hippocampal neuroinflammation. The left panel illustrates a homeostatic microenvironment in which resting microglia and supportive astrocytes maintain metabolic balance and preserve synaptic integrity. The right panel depicts chronic microglial activation and the release of IL‐1α, TNFα, and C1q, which drive astrocytes toward a neurotoxic A1 phenotype. This inflammatory shift disrupts mitochondrial stability and weakens neuronal resilience. To reflect downstream molecular consequences described in the manuscript, the figure highlights two significant signalling impairments: (i) PI3K/Akt downregulation, leading to mitochondrial instability and increased synaptic vulnerability; and (ii) MAPK/ERK dysregulation, which contributes to deficits in synaptic plasticity. Together, these convergent pathways exacerbate neuroinflammatory stress and heighten hippocampal susceptibility in neurodegenerative disorders.

Communication between microglia is fundamental for maintaining CNS homeostasis, and their interaction with astrocytes is equally critical for modulating both the initiation and resolution of immune responses.^4^ In the context of neurodegenerative diseases, however, these cells often enter a state of heightened activation, characterized by the excessive release of pro‐inflammatory mediators that contribute to the establishment of a chronic inflammatory environment.^3^ Upon activation, microglia release a trio of signalling molecules—IL‐1α, TNFα, and C1q—that drive astrocytes into a neurotoxic “A1” phenotype, which contributes to neuronal death (Figure 1).^13^ A recent study investigating microglia–astrocyte communication in murine models of neuronopathic mucopolysaccharidoses demonstrated that the induction of the A1 astrocyte phenotype contributes to the maintenance of neuroinflammation in neurodegenerative disorders.^14^

In response to this inflammatory stimulus, microglia undergo rapid phenotypic changes: their processes retract, the cells adopt an amoeboid shape, and they migrate toward sites of damage.^15^ At this stage, they actively phagocytose cellular debris, damaged neurons, and pathogens.^16^

Traditionally, microglial activation has been classified into two phenotypes: pro‐inflammatory (M1) and anti‐inflammatory (M2).^16^, ^17^ The M1 phenotype is associated with the release of cytokines and mediators that amplify inflammation and promote neurotoxicity, whereas the M2 phenotype suppresses inflammation and fosters tissue repair.^18^ However, this binary classification is now recognized as overly simplistic, as it does not adequately capture the diversity and complexity of microglial responses.^11^

Microglia are remarkably dynamic cells that constantly monitor the brain parenchyma through their highly motile processes. They maintain continuous activity, transitioning between diverse functional states and adjusting their responses according to environmental signals under both physiological and pathological conditions.^16^

During neurodegenerative processes, microglia display a continuum of mixed or transitional phenotypes rather than neatly fitting into the traditional M1/M2 categories, underscoring the oversimplification of this binary model in capturing the complexity of microglial activation.^11^

Microglia and astrocytes play crucial and interconnected roles in regulating synaptic plasticity and maintaining the structural integrity of neural circuits.^19^ Microglia dynamically regulate synapse formation, remodelling, and elimination during both development and adulthood.^20^ Complement cascade proteins, such as C1q and C3, which are widely expressed in the developing brain, label subsets of immature synapses for elimination. These tagged synapses are phagocytosed by microglia via the complement receptor C3 (C3R) pathway, ensuring precise synaptic refinement.^21^

Beyond synaptic pruning, microglia also promote synaptic strengthening through the phosphatidylinositol 3‐kinase (PI3K)/BDNF signalling pathway. Brain‐derived neurotrophic factor (BDNF), acting downstream of PI3K activation, enhances dendritic spine plasticity and connectivity in the adult cortex.^22^

Astrocytes similarly modulate synaptic activity and plasticity through the release of neurotrophic molecules such as BDNF, glial cell line‐derived neurotrophic factor (GDNF),^23^ and insulin‐like growth factor 1 (IGF‐1).^24^ These factors support neuronal survival, axonal growth, and synaptic maturation.^25^ However, during neuroinflammation, astrocytes can undergo a reactive transformation, disrupting these homeostatic processes. As mentioned previously, the A1 astrocyte phenotype, induced by microglia‐derived IL‐1α, TNFα, and C1q, loses its neuroprotective functions and secretes neurotoxic and pro‐inflammatory mediators that exacerbate neuronal damage.^13^

Thus, the crosstalk between microglia and astrocytes plays a decisive role in determining whether the synaptic environment favours repair and plasticity or contributes to chronic neurodegeneration.^4^, ^21^

Although A1 astrocytes play a prominent role in chronic neurodegenerative settings, astrocytic reactivity encompasses a broader spectrum.^13^ Under specific contexts—particularly acute CNS injury—astrocytes may adopt an A2 phenotype, characterized by the upregulation of neurotrophic and synaptogenic factors that support neuronal survival and tissue repair.^26^ This A1–A2 continuum underscores the functional plasticity of astrocytic responses and highlights that reactive astrocytes are not uniformly detrimental, but rather context‐dependent modulators of neuroinflammatory signalling.^27^, ^28^

While chronic neuroinflammation is a major contributor to synaptic dysfunction,^13^, ^21^ it is only one of several converging mechanisms underlying neuronal impairment. Another critical and tightly interconnected process involves mitochondrial dysfunction, which disrupts neuronal bioenergetics and calcium homeostasis.^29^ Given that synaptic transmission is highly energy‐dependent, mitochondrial dysfunction profoundly impairs synaptic maintenance and plasticity.^30^

### 2.2 Mitochondrial dysfunction and bioenergetic failure in synaptic degeneration

Mitochondrial dysfunction plays a central role in the progressive loss of synapses, as mitochondria are essential for local energy supply and calcium buffering required for neurotransmission.^30^, ^31^ They function as the central hubs of cellular energy metabolism, generating ATP and nicotinamide adenine dinucleotide, both of which are essential for reestablishing ionic gradients across neuronal membranes during synaptic signalling.^32^ This bioenergetic support is especially critical within axons and dendrites, where mitochondria not only sustain energy supply but also regulate intracellular calcium dynamics—a fundamental process for synaptic transmission, modulation, and plasticity.^33^, ^34^

Factors such as oxidative stress, impaired energy metabolism, failure of mitochondrial quality‐control pathways, such as mitophagy, and dysregulation of calcium homeostasis contribute to this dysfunction.^29^

These alterations preceded classical pathological events, as in Alzheimer's disease, including β‐amyloid plaque deposition and Tau hyperphosphorylation, both of which further aggravate mitochondrial impairment and oxidative burden.^35^ In addition to impairing synaptic efficiency, mitochondrial dysfunction compromises the integrity of the blood–brain barrier, facilitating the entry of neurotoxic substances into the brain parenchyma.^36^

**FIGURE 2:**
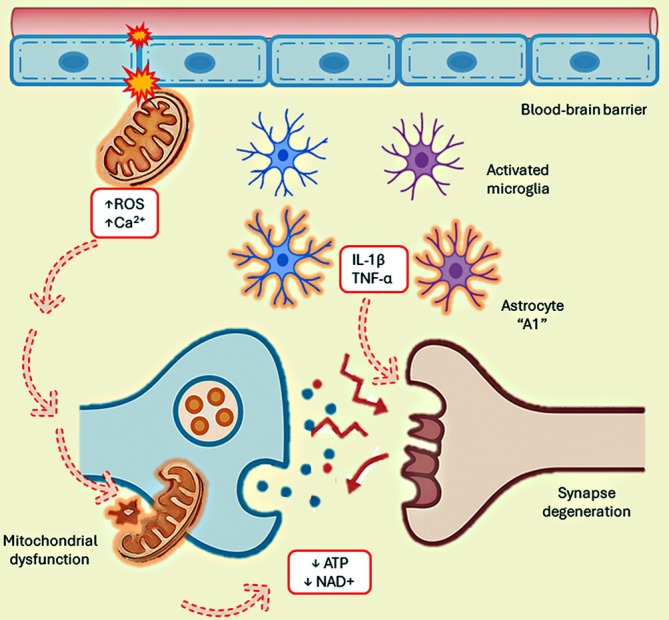
Mitochondrial dysfunction as a driver of blood–brain barrier disruption and synaptic degeneration. Mitochondrial impairment results in reduced ATP synthesis, nicotinamide adenine dinucleotide (NAD^+^) depletion, and excessive reactive oxygen species (ROS) production, leading to oxidative stress and dysregulation of calcium signalling. These alterations disrupt neuronal energy homeostasis and impair synaptic transmission and plasticity. Concurrently, mitochondrial dysfunction promotes blood–brain barrier breakdown by inducing endothelial oxidative injury and tight junction disassembly, facilitating the entry of neurotoxic molecules into the brain parenchyma. The combined bioenergetic collapse and barrier disruption trigger chronic cellular stress, neuronal injury, and progressive synaptic loss—key pathological features of neurodegenerative diseases such as Alzheimer's disease.

The resulting decrease in ATP production, increased generation of reactive oxygen species (ROS), and calcium imbalance collectively induce cellular stress and neuronal damage, ultimately leading to synaptic degeneration (Figure 2).^35^

Mitochondrial dysfunction not only compromises synaptic bioenergetics but also integrates with systemic metabolic alterations that drive neuroinflammation. Like the hypothalamus, the hippocampus is also insulin‐sensitive, and disruptions in insulin signalling pathways impair mitochondrial structure and integrity in both hippocampal and cortical neurons.^30^, ^37^ This convergence between impaired mitochondrial metabolism and insulin resistance establishes a key mechanistic link between peripheral metabolic stress and central neurodegenerative processes.^35^

### 2.3 Systemic insulin resistance and chronic inflammation in neuroinflammatory progression

Although traditionally regarded as a peripheral metabolic hormone, insulin also plays a fundamental neuromodulatory role in the brain, where its receptors are widely expressed in regions such as the hippocampus, cortex, and hypothalamus.^38^ Neurons are exquisitely sensitive to physiological insulin concentrations, and activation of the insulin receptor triggers a signalling cascade essential for neuronal survival, synaptic plasticity, and energy homeostasis.^37^

Systemic insulin resistance and chronic low‐grade inflammation create a permissive milieu for neuroinflammation in the central nervous system.^39^ Impaired insulin signalling in neurons and glial cells disrupts glucose utilization, compromises mitochondrial oxidative efficiency, and enhances ROS production.^37^, ^40^ Concomitantly, circulating pro‐inflammatory cytokines such as tumour necrosis factor‐α (TNF‐α), interleukin‐6 (IL‐6), and interleukin‐1β (IL‐1β) can cross the blood–brain barrier (BBB) or alter its integrity, thereby promoting microglial activation and astrocytic reactivity.^41^, ^42^ This metabolic‐inflammatory interplay amplifies neuroimmune signalling, culminating in synaptic dysfunction, neuronal energy failure, and progressive loss of structural plasticity—hallmarks of insulin‐resistant neurodegeneration.^21^

Moreover, insulin resistance in the brain often coexists with impaired insulin‐like growth factor 1 (IGF‐1) signalling, given the extensive molecular overlap between their receptors and downstream pathways. Both insulin and IGF‐1 receptors activate similar intracellular cascades, notably the PI3K/Akt and MAPK/ERK pathways, which regulate neuronal metabolism, survival, and synaptic maintenance.^43^, ^44^

Under insulin‐resistant conditions, diminished IGF‐1 activity exacerbates neuronal energy deficits and promotes a pro‐inflammatory phenotype in glial cells.^45^ Reduced IGF‐1 signalling also impairs the expression of anti‐inflammatory mediators and antioxidant enzymes, potentiating oxidative stress and microglial activation.^46^

Conversely, adequate IGF‐1 availability exerts neuroprotective effects by enhancing mitochondrial biogenesis, stabilizing synaptic proteins, and attenuating NF‐κB‐dependent inflammatory signalling.^44^

**FIGURE 3:**
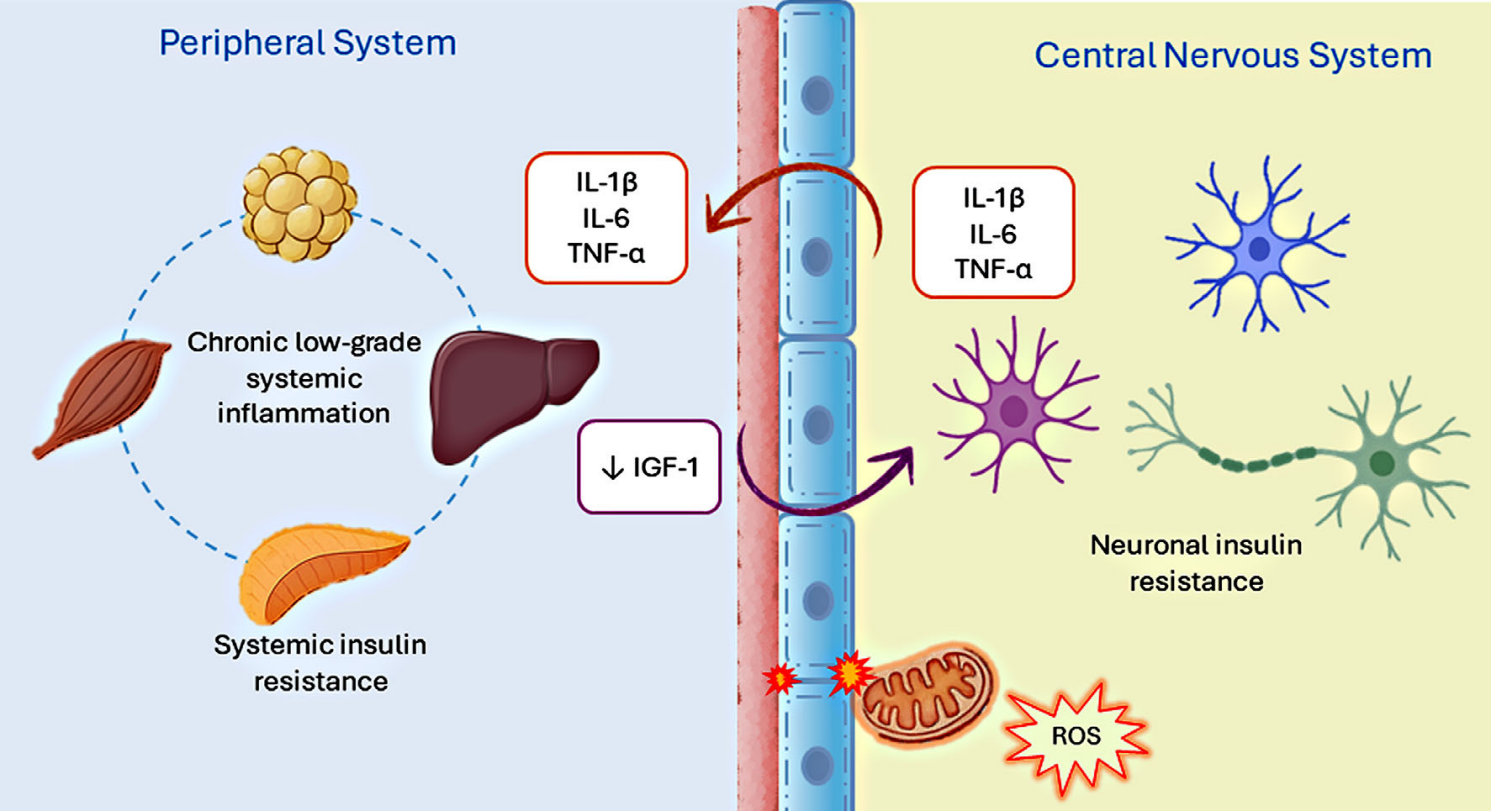
Systemic insulin resistance and chronic inflammation in the progression of neuroinflammation. The diagram illustrates the interplay between insulin resistance and low‐grade systemic inflammation in neuroenergetic dysfunction. Reduced insulin and IGF‐1 signalling impairs the PI3K/Akt and MAPK/ERK pathways, compromising mitochondrial homeostasis and synaptic plasticity. Circulating pro‐inflammatory cytokines (TNF‐α, IL‐6, and IL‐1β) can cross the blood–brain barrier or destabilize it, activating microglia and astrocytes. The loss of IGF‐1–mediated neuroprotection aggravates oxidative stress and glial inflammatory responses, establishing a self‐perpetuating cycle of metabolic disturbance and neuroinflammation that culminates in neuronal degeneration.

Together, impaired insulin and IGF‐1 signalling establish a chronic metabolic‐inflammatory milieu that disrupts neuronal energy metabolism and redox homeostasis.^39^ The resulting bioenergetic imbalance sensitizes neurons and glial cells to oxidative damage and inflammatory cues, perpetuating mitochondrial dysfunction and impairing synaptic integrity (Figure 3).^47^ Over time, this dual metabolic and inflammatory stress drives the transition from reversible signalling alterations to irreversible neurodegenerative processes. These convergent mechanisms bridge systemic metabolic disorders with central synaptic pathology, providing a mechanistic continuum toward insulin‐resistant neurodegeneration.^38^

## 3 EXPERIMENTAL MODELS OF NEUROINFLAMMATION

The elucidation of cellular and molecular pathways involved in neuroinflammation has advanced through the development of experimental models that reproduce distinct aspects of neurological disorders associated with inflammatory processes. The selection of an appropriate model is pivotal, as it defines not only the translational relevance of the findings but also the capacity to interrogate specific phases of the inflammatory cascade—from early microglial activation to progressive synaptic and neuronal degeneration.^48^, ^49^

This overview provides the experimental context necessary to interpret the mechanistic and translational findings discussed in subsequent sections. Genetically engineered models have provided critical insights into disease‐related inflammatory pathways. Transgenic lines overexpressing proteins such as amyloid precursor protein/presenilin‐1 (APP/PS1) or Tau‐P301S or carrying deletions in innate immunity regulators like triggering receptor expressed on myeloid cells 2 (TREM2) and NOD‐, LRR‐, and pyrin domain‐containing protein 3 (NLRP3), have been instrumental in dissecting glial and neuronal responses within the inflamed brain.^50^, ^51^, ^52^, ^53^, ^54^

In contrast, metabolic induction models in wild‐type C57BL/6 mice—typically fed a high‐fat diet—have illuminated the systemic contribution of insulin resistance and mitochondrial dysfunction to neuroinflammatory processes.^39^, ^55^, ^56^

Complementary to these paradigms, intracerebroventricular (ICV) models employing lipopolysaccharide (LPS),^57^ β‐amyloid oligomers,^58^ or streptozotocin^59^ can induce acute or chronic central inflammation independently of peripheral metabolic cues.

Because many studies in the field use LPS‐ICV to characterize anti‐inflammatory actions of GLP‐1RA—despite its disproportionately acute inflammatory profile—we provide a critical evaluation of this model to prevent overestimating the translational value of findings generated under these conditions.

It is important to acknowledge LPS‐ICV limitations within the context of chronic neurodegenerative disease. Although LPS models are valuable for probing acute microglial activation, cytokine signalling, and mitochondrial stress responses, LPS administration triggers a rapid and supra‐physiological TLR4‐driven inflammatory cascade that does not mirror the low‐grade, sustained inflammation characteristic of Alzheimer's disease.^60^ Moreover, chronic TLR4 stimulation fails to induce progressive protein aggregation, as demonstrated by studies showing no enhancement of Tau pathology despite persistent microgliosis.^61^, ^62^ These models also lack chronic metabolic impairment, including insulin resistance and long‐term bioenergetic decline, which represent central drivers of neurodegenerative vulnerability. Furthermore, LPS paradigms rarely reproduce prolonged glial remodelling, slow synaptic deterioration, or gradual mitochondrial dysfunction, all of which define the progressive trajectory of human Alzheimer's pathology.^63^ For these reasons, findings from acute LPS‐induced neuroinflammation should be interpreted primarily as mechanistic insights, and their translational relevance relies on convergence with complementary genetic, metabolic, and chronic inflammatory models that better capture the multidimensional progression of neurodegenerative disease.

At the in vitro level, cellular models—including primary neuronal and microglial cultures, immortalized lines such as SH‐SY5Y (a human neuroblastoma cell line),^64^, ^65^, ^66^ and BV2 (a murine microglial cell line),^67^ and human induced pluripotent stem cell (iPSC)‐derived brain organoids^68^—offer tractable systems for mechanistic dissection and pharmacological screening under controlled conditions.

Significantly, the mechanistic actions attributed to GLP‐1RA—modulation of microglial reactivity, mitochondrial stabilization, restoration of insulin signalling, and attenuation of synaptic stress—vary in consistency across the different inflammatory contexts simulated by each model. Acute LPS paradigms reveal rapid cytokine and TLR4‐dependent effects, whereas chronic metabolic and genetic models uncover slower‐acting pathways related to mitochondrial resilience and microglial remodelling. Recognizing these model‐specific contributions is therefore critical for interpreting the coherence of preclinical literature and for identifying which mechanisms are most likely to translate into human neurodegenerative disease.

**FIGURE 4:**
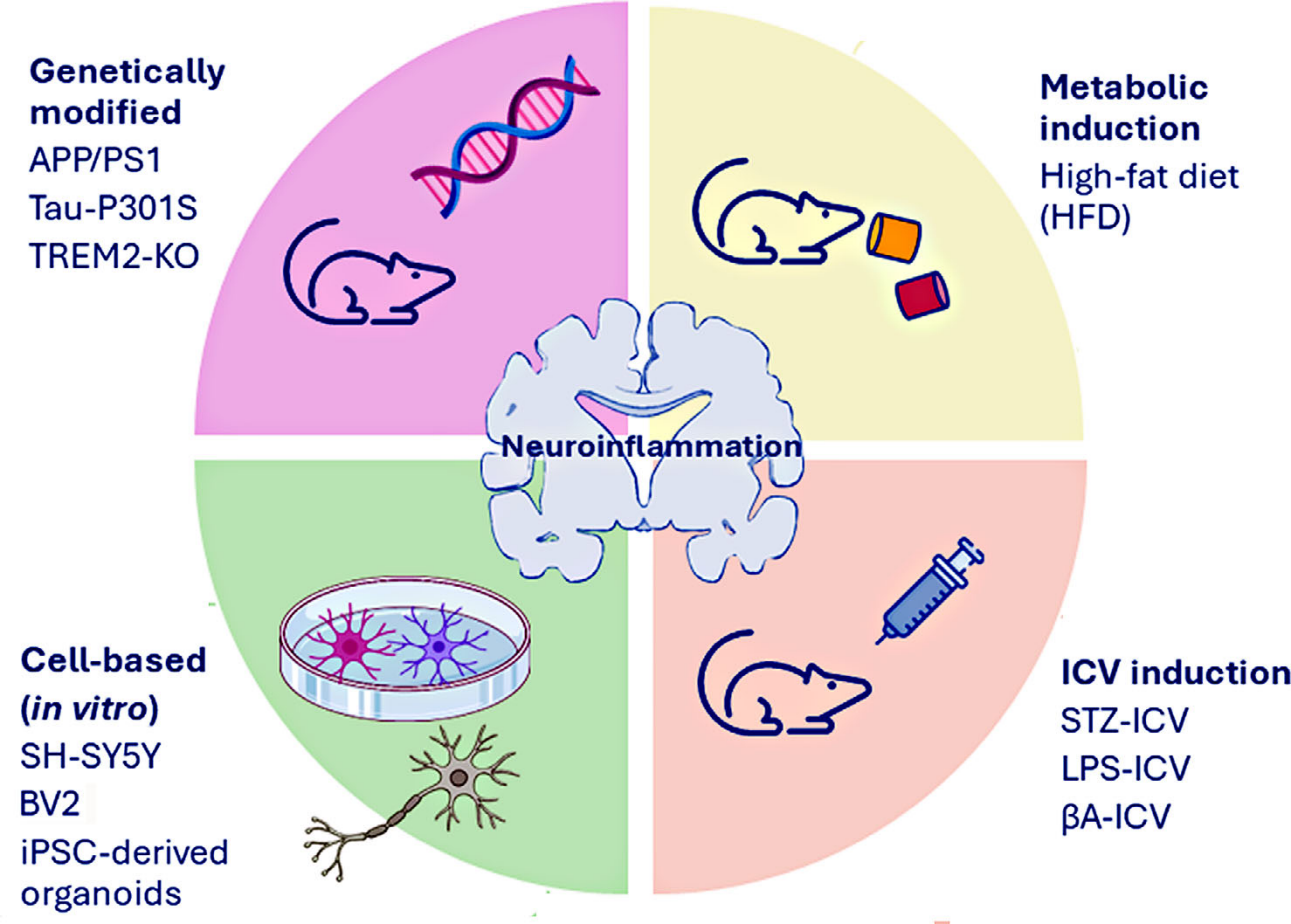
Experimental models used to study neuroinflammation. Neuroinflammatory processes are investigated through complementary experimental approaches. Genetically modified models (e.g., APP/PS1, TREM2 knockout) elucidate intrinsic glial and neuronal mechanisms. Metabolic models (e.g., high‐fat diet) establish links between systemic insulin resistance, mitochondrial dysfunction, and central inflammation. Intracerebroventricular (ICV) models using agents such as LPS or β‐amyloid allow controlled induction of central inflammation. Cellular systems (e.g., BV2, SH‐SY5Y, and iPSC‐derived organoids) offer simplified platforms for mechanistic and pharmacological exploration. Collectively, these models provide an integrative framework connecting molecular mechanisms to functional outcomes in neuroinflammatory progression.

Collectively, these complementary models provide a multifaceted framework for studying neuroinflammation, bridging molecular alterations with cellular dysfunction and behavioural outcomes (Figure 4 and Table 1).^69^ However, the intrinsic limitations of each system underscore the need for integrative experimental strategies that combine genetic, metabolic, and cellular approaches to better recapitulate the complexity of human neuroinflammatory disorders.

**TABLE 1:** Experimental models of neuroinflammation.

Model type	Representative example	Primary target/pathology	Induction time	Advantages	Limitations	Applicability to incretin‐based therapies	Key references
Genetically modified	APP/PS1, Tau‐P301S, TREM2‐KO	Aβ deposition, Tau aggregation, and microglial dysfunction	4–12 months	Stable phenotype, high reproducibility, human‐relevant mutations	High cost, limited metabolic context, slow onset	Limited utility for incretin studies; useful for microglial inflammation	^50^, ^51^, ^52^, ^53^, ^54^
Metabolic induction	HFD, HFD + STZ	Peripheral insulin resistance, systemic inflammation	8–16 weeks	High translational relevance, mimics diabetic milieu	Variable phenotype, moderate reproducibility	Highly suitable for evaluating GLP‐1/GIP‐based therapies	^39^, ^55^
Intracerebroventricular induction	STZ‐ICV, LPS‐ICV, βA‐ICV	Central insulin signalling impairment, microglial activation	2–4 weeks	Rapid induction, robust neuroinflammatory phenotype	Acute lesion, lacks peripheral metabolic features	Helpful in assessing the central neuroprotective effects of incretins	^57^, ^58^, ^59^
Cell‐based (in vitro)	SH‐SY5Y, BV2, iPSC‐derived organoids	Specific molecular and inflammatory pathways	Days‐weeks	High control, reproducibility, suitable for mechanistic and drug screening studies	Lacks systemic integration, vascular and immune interactions	Ideal for early‐phase testing and mechanistic validation of incretin effects	^64^, ^65^, ^66^, ^67^, ^68^

*Note*: These models are essential tools for dissecting the molecular and cellular mechanisms underlying the interplay among metabolic dysfunction, immune activation, and neurodegeneration. Each model system offers unique advantages and limitations, depending on the research objective, ranging from genetic predisposition to systemic metabolic perturbation to direct neuroinflammatory induction. The following table summarizes key characteristics of four major experimental categories, emphasizing their translational applicability for studying insulin resistance‐associated neuroinflammation and evaluating incretin‐based therapeutic strategies.

Abbreviations: APP/PS1, amyloid precursor protein/presenilin‐1; βA, beta‐amyloid oligomers; BV2, mouse microglial cell line; HFD, high‐fat diet; ICV, intracerebroventricular; iPSC, induced pluripotent stem cell; KO, knockout; LPS, lipopolysaccharide; SH‐SY5Y, human neuroblastoma cell line; STZ, streptozotocin; Tau, microtubule‐associated protein Tau; TREM2, triggering receptor expressed on myeloid cells 2.

## 4 INCRETIN‐BASED THERAPEUTIC STRATEGIES IN NEUROINFLAMMATION AND METABOLIC DYSREGULATION

Incretins, such as glucagon‐like peptide‐1 (GLP‐1) and glucose‐dependent insulinotropic polypeptide (GIP), are gut‐derived hormones that potentiate glucose‐stimulated insulin secretion, thereby playing a crucial role in maintaining systemic energy homeostasis.^70^, ^71^ Beyond their classical endocrine actions in the pancreas, incretins exert pleiotropic effects on peripheral tissues—including the liver, adipose tissue, and skeletal muscle—and have emerged as modulators of inflammation, oxidative stress, and mitochondrial function.^72^, ^73^ These wide‐ranging actions have prompted growing interest in their potential to counteract neuroinflammatory and neurodegenerative processes associated with metabolic dysfunction.

### 4.1 Neuroprotective and anti‐inflammatory mechanisms of incretins

GLP‐1 receptors (GLP‐1R) are expressed not only in pancreatic β‐cells but also in neurons, astrocytes, and microglia across several brain regions, including the hippocampus, cortex, and hypothalamus.^74^ Activation of these receptors triggers intracellular cascades, including PI3K/Akt, cAMP/PKA, and MAPK/ERK pathways, which converge to regulate mitochondrial biogenesis, synaptic maintenance, and redox balance.^75^ In neuronal and glial populations, GLP‐1R stimulation promotes the expression of neurotrophic factors like BDNF and IGF‐1, enhances autophagy and mitophagy, and suppresses pro‐inflammatory transcriptional programs mediated by nuclear factor kappa‐light‐chain‐enhancer of activated B cells (NF‐κB).^9^

Experimental and preclinical evidence demonstrates that GLP‐1RA, including exendin‐4 and liraglutide, exerts potent neuroprotective^76^, ^77^ and anti‐inflammatory actions within the central nervous system.^78^, ^79^ GLP‐1R activation attenuates microglial reactivity and suppresses the release of pro‐inflammatory cytokines such as TNF‐α and IL‐1β, thereby dampening neuroinflammatory cascades.^9^, ^80^ Central GLP‐1R activation also suppresses TLR‐driven cytokine induction (e.g., TNF‐α) via neuronal GLP‐1Rs, indicating a direct central mechanism beyond peripheral metabolic effects.^81^

At the cellular level, GLP‐1R stimulation restores mitochondrial membrane potential, enhances ATP generation, and promotes mitophagy, thereby improving neuronal bioenergetics and redox balance.^82^ In models of neurodegeneration such as APP/PS1 mice, liraglutide treatment normalizes synaptic markers—including PSD‐95 and synaptophysin—and increases dendritic spine density, correlating with improved cognitive performance.^83^, ^84^ Collectively, these findings indicate that incretin signalling modulates the metabolic‐inflammatory interface of the brain, restoring homeostatic equilibrium under insulin‐resistant or neuroinflammatory conditions. To provide a consolidated overview of these documented neuroprotective mechanisms, the main CNS‐relevant effects of GLP‐1RA are summarized in Table 2.

**TABLE 2:** Documented CNS effects of GLP‐1 receptor agonists.

Category	Effect	Representative evidence	Key references
Neuroinflammation	↓ TNF‐α/IL‐1β/IL‐6	GLP‐1RAs reduce pro‐inflammatory cytokines in the hippocampus and cortex	^79^, ^81^, ^92^, ^105^, ^127^
Neuroinflammation	↓ Microglial activation (↑CD206, ↑Arg1, ↓iNOS)	Exenatide and liraglutide induce M2‐like microglial shift	^92^, ^105^, ^126^, ^127^
Synaptic plasticity	↑ PSD‐95	Liraglutide and exendin‐4 rescue synaptic density in AD models	^75^, ^84^
Synaptic plasticity	↑ Synaptophysin	GLP‐1RAs preserve presynaptic terminals in neurodegeneration	^84^, ^96^
Mitochondrial function	↑ PGC‐1α/biogenesis	GLP‐1RAs enhance mitochondrial biogenesis via PKA–Epac–PI3K pathways	^82^, ^86^, ^94^
Mitochondrial Function	↓ Oxidative stress	Semaglutide and exendin‐4 improve mitochondrial stability and reduce ROS	^89^, ^95^
Neurotrophic Support	↑ BDNF	Liraglutide and exendin‐4 increase BDNF and support neuronal survival	^84^, ^89^, ^96^

Abbreviations: AD, Alzheimer's disease; AMPK, AMP‐activated protein kinase; Arg1, Arginase‐1; BDNF, Brain‐derived neurotrophic factor; BBB, Blood–brain barrier; CNS, Central nervous system; CD206; Mannose receptor C‐type 1; GLP‐1RA, Glucagon‐like peptide‐1 receptor agonist; GIP, Glucose‐dependent insulinotropic polypeptide; iNOS, Inducible nitric oxide synthase; IL‐1β, Interleukin‐1 beta; IL‐6, Interleukin‐6; LPS, Lipopolysaccharide; NF‐κB, Nuclear factor kappa‐light‐chain‐enhancer of activated B cells; PGC‐1α, Peroxisome proliferator–activated receptor gamma coactivator 1‐alpha; PKA, Protein kinase A; PSD‐95, Postsynaptic density protein 95; ROS, Reactive oxygen species; TNF‐α, Tumour necrosis factor alpha.

#### 4.1.1 Direct CNS actions of GLP‐1RA

A growing body of evidence demonstrates that GLP‐1RA exerts direct, cell‐autonomous effects within the central nervous system, beyond their systemic metabolic actions. Microglia express components of the GLP‐1 signalling machinery and respond directly to GLP‐1R stimulation, displaying reduced TNF‐α and IL‐1β release in both primary and immortalized cultures.^72^, ^85^ Central GLP‐1R activation also suppresses TLR4‐driven cytokine induction via neuronal GLP‐1Rs, further supporting a direct CNS mechanism independent of improvements in peripheral glucose homeostasis.^81^ At the intracellular level, GLP‐1R engagement activates cAMP/PKA^86^ and Epac→PI3K/Akt cascades,^82^, ^87^ while inhibiting NF‐κB^81^ and MAPK pathways^88^, ^89^ —molecular programs that enhance mitophagy, improve mitochondrial membrane potential, and promote mitochondrial quality control. Collectively, these mechanistic insights indicate that incretin mimetics modulate central neuroimmune and bioenergetic pathways directly within neurons and microglia, providing a biological basis for their disease‐modifying potential in neurodegenerative disorders.

### 4.2 LPS‐ICV model as a tool to investigate incretin‐mediated neuroprotection

The intracerebroventricular lipopolysaccharide (LPS‐ICV) model represents a robust approach to studying neuroinflammation in the absence of systemic confounders.^57^ In this paradigm, LPS—a potent Toll‐like receptor 4 (TLR4) agonist—is administered directly into the lateral ventricle, eliciting a controlled neuroimmune response characterized by microglial and astrocytic activation, elevated cytokine production, and neuronal loss, particularly within the hippocampus.^90^ This model reproduces key molecular features observed in early neurodegenerative states, including oxidative stress, mitochondrial fragmentation, and synaptic dysfunction.^91^

GLP‐1RAs have been extensively evaluated in experimental models of neuroinflammation. Administration of liraglutide or exendin‐4 markedly attenuates microglial activation. It decreases hippocampal levels of pro‐inflammatory cytokines such as TNF‐α, IL‐1β, and IL‐6, while concurrently inhibiting NF‐κB nuclear translocation and promoting an anti‐inflammatory microglial phenotype.^92^ These effects have been documented in models employing ICV or systemic LPS administration, which typically induces robust neuroinflammatory responses.^93^

Furthermore, liraglutide and exendin‐4 enhance mitochondrial biogenesis by upregulating PGC‐1α, thereby improving mitochondrial quality control.^94^ They also restore the expression of synaptic proteins such as PSD‐95 and synaptophysin, thereby supporting synaptic integrity and cognitive performance.^95^ In line with these findings, exendin‐4 treatment prevents neuronal loss and ameliorates spatial memory and anxiety‐like behaviours in rodent models of sporadic dementia.^96^

Of note, the neuroprotective efficacy of incretin analogs in LPS‐ICV paradigms appears to be partially independent of their peripheral metabolic effects, underscoring their direct central actions on glial signalling, oxidative stress, and neuronal resilience.^97^ Collectively, these findings support the concept that incretin‐based therapies function as metabolic–neuroimmune modulators, bridging peripheral insulin sensitivity with CNS homeostasis.

### 4.3 Integration with insulin and IGF‐1 signalling pathways

Given the substantial molecular overlap between incretin receptor signalling and the insulin/IGF‐1 axis, restoration of GLP‐1R activity may compensate for deficits in the PI3K/Akt and MAPK/ERK cascades that characterize insulin‐resistant neurodegeneration.^39^ Incretins not only enhance neuronal glucose utilization and mitochondrial efficiency but also attenuate the pro‐inflammatory milieu that perpetuates chronic glial activation.^98^

Although GLP‐1R and IGF‐1R are distinct receptors, their intracellular signalling cascades converge on the PI3K–Akt and MAPK/ERK pathways, which regulate neuronal survival, mitochondrial dynamics, and synaptic plasticity.^99^ Impaired insulin/IGF‐1 signalling—characteristic of T2DM and Alzheimer's disease—reduces Akt activity, disrupts mitochondrial homeostasis, and promotes oxidative vulnerability.^9^ Preclinical studies show that GLP‐1RA can partially compensate for these deficits by enhancing Akt phosphorylation, restoring mitochondrial membrane potential, and attenuating pro‐inflammatory signalling in insulin/IGF‐1‐resistant models.^100^, ^101^ Importantly, GLP‐1RA does not directly activate IGF‐1 receptors but reinforces downstream metabolic and cytoprotective nodes commonly sustained by insulin/IGF‐1 signalling.^102^, ^103^ This indirect convergence provides a mechanistic rationale for their capacity to counteract the feed‐forward cycle linking insulin/IGF‐1 resistance, mitochondrial dysfunction, and neuroinflammation.

Experimental studies have shown that central activation of GLP‐1R reduces systemic inflammatory responses, such as TNF‐α induction by Toll‐like receptor agonists, via neuronal signalling (independent of haematopoietic or endothelial GLP‐1R).^81^ This mechanism demonstrates a brain–gut GLP‐1R axis that modulates peripheral inflammation, reinforcing the role of GLP‐1 in the integrated regulation of metabolic stress and neuroinflammation.

Through these convergent mechanisms, incretin‐based therapies may disrupt the feed‐forward cycle linking mitochondrial dysfunction, metabolic stress, and glial activation—a core pathogenic axis shared by T2DM and Alzheimer's disease.^104^

### 4.4 Preclinical evidence and translational perspectives

Preclinical studies have provided consistent evidence that incretin‐based therapies can modulate key mechanisms implicated in neurodegenerative processes, including neuroinflammation, mitochondrial dysfunction, and synaptic resilience. A broad spectrum of incretin mimetics—ranging from classical GLP‐1RAs to dual GLP‐1/GIP receptor agonists and emerging triagonists—has been developed and evaluated across diverse experimental paradigms.^98^ Their main pharmacological and neuroprotective characteristics are summarized in Table 3, which highlights receptor selectivity, molecular structure, and documented effects on metabolic and neuronal endpoints.

**TABLE 3:** Chronological evolution of incretin‐based therapies, receptor specificity, clinical stage, and reported neuroprotective mechanisms.

Generation/agent	Receptor targets	Developer/manufacturer	Administration route	Key approvals/stage	Year (approx.)	BBB penetration/CNS effects	Key references
Exenatide	GLP‐1	Amylin/AstraZeneca	Subcutaneous	Approved for T2DM	2005	Limited BBB penetration; modest neuroprotective data in AD models. (*)	^105^, ^106^
Liraglutide	GLP‐1	Novo Nordisk	Subcutaneous	Approved for T2DM, obesity	2010	Crosses BBB; improves cognition and reduces neuroinflammation in LPS and AD models. (***)	^107^, ^109^, ^110^, ^111^, ^113^
Lixisenatide	GLP‐1	Sanofi	Subcutaneous	Approved for T2DM	2013	Demonstrated BBB penetration and neuroprotective properties in preclinical models. (***)	^108^, ^112^
Semaglutide	GLP‐1	Novo Nordisk	Subcutaneous / Oral	Approved for T2DM, obesity; trials in AD (EVOKE)	2017–2019	Likely limited BBB penetration; indirect neuroprotective mechanisms under investigation. (**)	^74^, ^114^, ^115^, ^116^, ^117^
Cotadutide	GLP‐1 + Glucagon	AstraZeneca	Subcutaneous	Phase II (NASH, obesity)	2020s	Improves hepatic lipid metabolism; potential indirect CNS benefit. (*)	^118^, ^138^, ^139^
Tirzepatide	GLP‐1 + GIP	Eli Lilly	Subcutaneous	Approved for T2DM (SURPASS), obesity (SURMOUNT)	2022	Reduces neuroinflammatory markers and oxidative stress in preclinical models. (**)	^119^, ^120^
Retatrutide	GLP‐1 + GIP + Glucagon	Eli Lilly	Subcutaneous	Phase III (obesity, diabetes)	2023–2024	Strong systemic efficacy; CNS effects under evaluation. (**)	^122^, ^140^

*Note*: BBB penetration and CNS effects are based on available preclinical and clinical evidence. BBB penetration ratings refer to both direct parenchymal entry and indirect CNS engagement at the blood–brain barrier interface (e.g., endothelial or circumventricular signalling). For semaglutide, available data support limited parenchymal access but preserved central actions through noncanonical routes. Only a single experimental study has reported potential anti‐inflammatory or neuroprotective effects of tirzepatide. In contrast, a rigorous investigation in 5XFAD and APP/PS1 mice found no improvement in pathology, neuroinflammation, behaviour, or cognition after treatment with semaglutide or tirzepatide.^120^ Evidence levels are indicated qualitatively as robust (***), moderate (**), or limited (*). Accordingly, tirzepatide was assigned “moderate evidence (++)” due to the sparse, heterogeneous nature of the available data.

Abbreviations: BBB, blood–brain barrier; CNS, central nervous system; GLP‐1, glucagon‐like peptide‐1; GIP, glucose‐dependent insulinotropic polypeptide; T2DM, type 2 diabetes mellitus; NASH, non‐alcoholic steatohepatitis.

Exenatide shows limited BBB penetration but demonstrates modest neuroprotective activity in rodent models of spinal cord injury, with improvements in microglial polarization and ER‐stress markers.^105^, ^106^

Liraglutide and lixisenatide have the strongest CNS evidence: both cross the BBB in animal studies and consistently improve cognition, neuroinflammation, and neurogenesis in transgenic and Alzheimer's models,^107^, ^108^, ^109^, ^110^ with liraglutide additionally preserving cerebral glucose metabolism in a controlled clinical trial.^111^, ^112^, ^113^

While these studies primarily address mechanisms relevant to progressive neurodegenerative processes, additional evidence comes from ischemia–reperfusion models, which reveal a mechanistically distinct, acute form of GLP‐1–mediated neuroprotection. These models showed that GLP‐1 receptor agonists exert robust acute neuroprotective effects, particularly in reducing infarct volume and early functional deterioration. In rats subjected to middle cerebral artery occlusion, both liraglutide and semaglutide markedly reduced infarct size when administered at the onset of reperfusion, an effect linked to direct GLP‐1 receptor activation and attenuated by receptor blockade. These findings highlight a mechanism primarily related to acute cytoprotection in ischemic injury, which differs conceptually from progressive neurodegenerative processes such as Alzheimer's disease.^76^

Semaglutide shows mixed BBB evidence, with likely restricted parenchymal entry, but documented preclinical studies of cognitive and neuroimmune benefits attributed to circumventricular access and systemic anti‐inflammatory effects,^114^, ^115^, ^116^ consistent with emerging data from EVOKE/EVOKE+.^74^, ^117^ Given the relevance of EVOKE/EVOKE+ as the first large‐scale Phase 3 trials of semaglutide in Alzheimer's disease, we now provide a more detailed contextualization. These studies are designed to evaluate cognitive decline and biomarker trajectories over extended follow‐up in early AD; at the present stage, only preliminary safety and feasibility data are available, with no confirmed efficacy outcomes. Their ongoing status underscores both the clinical momentum and the current evidentiary gap.

Evidence for cotadutide remains indirect, primarily reflecting improvements in systemic lipid and inflammatory pathways.^118^

Tirzepatide shows preliminary evidence of neuroprotective actions, including reduced oxidative stress and microglial activation, though direct BBB penetration has not been confirmed.^119^ However, the overall evidence for tirzepatide remains limited and heterogeneous. While one study has reported reductions in oxidative stress and microglial activation, a recent high‐quality investigation in 5XFAD and APP/PS1 mice found no improvement in neuropathology, neuroinflammation, behaviour, or cognitive outcomes following treatment with semaglutide or tirzepatide.^120^ In line with these mixed findings, the evidence level for tirzepatide in Table 3 has been revised to ‘moderate’ (++).

Retatrutide's CNS activity is still under evaluation, with no current evidence of BBB entry.^121^, ^122^ These distinctions form the basis for the qualitative evidence levels assigned in Table 2.

Collectively, these compounds delineate a pharmacological continuum linking peripheral metabolic regulation to central neuroimmune homeostasis, thereby setting the stage for translational exploration in neurodegenerative disorders.^123^ Beyond agent‐specific profiles, preclinical evidence clusters into mechanistic domains that map directly onto principal pathological axes of neurodegeneration.

The largest body of evidence addresses AD‐related amyloid pathology. Multiple studies demonstrate that liraglutide and semaglutide attenuate amyloid‐β deposition, reduce soluble oligomers, preserve synaptic plasticity, and improve memory performance in APP/PS1 and 3xTg‐AD mice. Liraglutide reliably prevents synapse loss, restores long‐term potentiation, and reduces microglial activation even after plaques are established.^77^, ^84^, ^109^ Semaglutide expands this profile by improving behavioural outcomes and altering amyloid load in both mice and human AD brain organoids, while also increasing oxytocin levels—suggesting additional neuromodulatory effects.^124^ This cluster positions GLP‐1 agonists within a broad therapeutic context of amyloid‐driven pathology and synaptic resilience.

Beyond amyloid pathology, a prominent mechanistic axis involves GLP‐1 agonists' regulation of microglial activation and neuroinflammatory signalling. Several studies converge on anti‐inflammatory mechanisms, highlighting GLP‐1 agonists as regulators of microglial activation states and inflammatory cascades. Liraglutide reduces chronic inflammation induced by irradiation, lowering cytokine levels (IL‐6, IL‐12p70, IL‐1β) and decreasing activated microglia and astrocytes in cortical and hippocampal regions.^125^ Semaglutide promotes microglial polarization from pro‐inflammatory M1 to M2 phenotypes, suppresses TLR4/NF‐κB signalling, reduces amyloid burden, and restores cognitive function.^53^, ^54^ These findings underscore the role of GLP‐1 agonists as modulators of neuroimmune tone in both acute and chronic neurodegenerative settings.

Mechanistic investigations in transgenic and toxin‐induced models of Alzheimer's and Parkinson's diseases have demonstrated that GLP‐1R activation attenuates microglial activation, suppresses NF‐κB signalling, and restores mitochondrial biogenesis and ATP production.^74^ Studies investigating the effects and mechanisms of GLP‐1R activation by liraglutide and exendin‐4 have shown that GLP‐1R stimulation promotes an anti‐inflammatory microglial phenotype, characterized by increased mannose receptor C‐type 1 (CD206) and arginase 1 (Arg‐1) and reduced inducible nitric oxide synthase (iNOS).^105^, ^125^, ^126^, ^127^ This polarization is at least partly mediated via cAMP/PKA signalling, a canonical downstream pathway of GLP‐1R activation that has been implicated in suppressing NF‐κB activity and dampening pro‐inflammatory transcriptional programs.^92^, ^128^ These effects translate into reduced synaptic loss and improved cognitive performance, suggesting that incretin signalling contributes to neuronal survival through both anti‐inflammatory and bioenergetic pathways.^98^

A second group of studies converges on the ability of GLP‐1R agonists—especially liraglutide and semaglutide—to modulate autophagy flux, restore ER proteostasis, and suppress apoptotic signalling in neuronal or neuroblastoma cell models. Liraglutide rescues cells from chronic endoplasmic reticulum (ER) stress (e.g., thapsigargin‐induced) by normalizing ER stress proteins, restoring autophagic machinery, and engaging Akt/STAT3‐dependent survival signalling. Semaglutide counters amyloid‐β toxicity by enhancing autophagy markers (LC3‐II, Atg7, Beclin‐1, p62) while simultaneously reducing pro‐apoptotic Bax and restoring Bcl‐2 levels, indicating coordinated activation of cell survival pathways. These mechanistic studies align with a cellular protection narrative rather than a large‐scale tissue reorganization narrative.^66^, ^114^

A distinct cluster of preclinical studies focuses on tau‐driven neurodegeneration. In transgenic tauopathy models, liraglutide significantly reduces pathology‐specific tau phosphorylation, mitigates neurofibrillary tangle formation, and improves survival and motor outcomes, suggesting direct modulation of tau kinases such as GSK‐3β.^110^ Semaglutide extends these findings in P301S mice, where it enhances autophagic flux and regulates the ACE2–SIRT1–FOXO1 axis, ultimately decreasing tau propagation, microglial activation, and oxidative stress.^115^ These studies collectively frame GLP‐1 agonists as modulators of tau homeostasis and microglial reactivity within chronic neurodegenerative contexts.

Another cohesive group centers on metabolic regulation within the brain, particularly the GLP‐1R/SIRT1/GLUT4 axis. In 3xTg models, semaglutide enhances brain glucose utilization, increases SIRT1 and GLUT4 expression, improves glycolysis, and reverses metabolic dysfunction. These effects parallel cognitive improvements and reductions in amyloid and tau pathology. Mechanistically, semaglutide restores glucose transporter trafficking and promotes metabolic resilience, positioning impaired glucose metabolism as a pivotal target of GLP‐1–based neuroprotection.^116^

Dual GLP‐1/GIP receptor agonists —such as tirzepatide—further amplify these benefits, improving metabolic management in T2DM and obesity, including after menopause,^129^, ^130^ and suppressing oxidative stress and upregulating neurotrophic cascades compared to selective GLP‐1RAs.^119^

Beyond canonical incretin pathways, next‐generation co‐agonists targeting GLP‐1, GIP, and glucagon receptors (e.g., retatrutide) optimize systemic lipid metabolism and improve peripheral insulin sensitivity.^131^ These peripheral effects may secondarily influence central insulin signalling, highlighting the potential of multi‐receptor agonism to modulate metabolic, inflammatory, and mitochondrial dysfunction in neurodegenerative contexts.

From a translational standpoint, the growing convergence between metabolic and neurodegenerative research domains underscores the therapeutic promise of incretin‐based strategies.^98^ While clinical validation remains in early stages, the robust preclinical evidence supports the view that incretin mimetics act as metabolic–neuroimmune modulators, capable of restoring insulin signalling and dampening chronic neuroinflammation.^132^ Advancements in pharmacokinetic optimization—particularly in enhancing blood–brain barrier permeability—are expected to accelerate the transition of these agents from metabolic to neuroprotective indications.^123^

Collectively, these observations suggest that incretin‐based therapies occupy a unique position among disease‐modifying candidates for neurodegeneration, bridging peripheral and central mechanisms.

Complementing the robust preclinical evidence, retrospective cohort analyses have begun to reveal potential neuroprotective associations of GLP‐1RA in humans.^52^, ^133^ In an extensive multi‐institutional study including over 5 million obese adults, GLP‐1RA therapy (notably liraglutide, dulaglutide, and semaglutide) was associated with significantly lower risks of Alzheimer's disease, Parkinson's disease, and other neurodegenerative disorders, alongside reduced all‐cause mortality.^134^

Real‐world data support the translational potential suggested by animal studies. Large‐scale target‐trial emulation from US electronic health records shows significantly lower risk of Alzheimer's disease and AD‐related dementias in patients treated with semaglutide compared with insulin, metformin, and even older GLP‐1R agonists. Risk reductions persist across age, sex, and obesity subgroups, reinforcing the plausibility of semaglutide as a disease‐modifying agent in humans. Although they do not demonstrate causality, these studies provide compelling population‐level evidence for neuroprotective associations.^52^, ^133^

However, these analyses remain observational and therefore subject to limitations, including residual confounding, confounding by indication, and the inability to infer causality. Individuals receiving GLP‐1RAs may differ systematically from non‐users with respect to comorbidities, health‐seeking behaviour, and clinical monitoring. Thus, these findings indicate an association rather than direct neuroprotective effects.

Finally, the literature includes trial design work addressing how liraglutide might be tested in early Alzheimer's disease. Although it does not provide efficacy outcomes, the ELAD trial protocol outlines methodological considerations for evaluating GLP‐1RA effects in a clinical AD population, offering a bridge between mechanistic studies and therapeutic translation.^113^

## 5 CONCLUDING REMARKS

Collectively, the evidence discussed underscores the pivotal role of incretin signalling as a bridge between metabolic and neuroimmune homeostasis.^74^ Through GLP‐1R activation and its crosstalk with insulin and IGF‐1 pathways, incretin mimetics restore mitochondrial integrity, attenuate neuroinflammatory cascades, and preserve synaptic resilience.^135^ These mechanistic effects, consistently reproduced in experimental models, delineate a convergent framework in which a single pharmacological class can therapeutically target metabolic stress, oxidative injury, and glial activation.^81^, ^136^

The translational momentum gained from preclinical studies supports incretin‐based agents as promising candidates for disease modification in Alzheimer's disease and related disorders. As dual and triagonist formulations advance, capable of engaging multiple receptors along metabolic and neurotrophic axes, the field moves toward integrated therapeutic strategies that address the energetic, inflammatory, and degenerative dimensions of brain pathology.^137^ Despite these advances, significant challenges remain. Translational gaps between robust rodent models and heterogeneous human neurodegenerative disorders continue to limit predictive validity.^117^ Uncertainties regarding the optimal dose, treatment duration, and timing for neuroprotection relative to metabolic control also persist. In frail, elderly populations, risks such as gastrointestinal intolerance, dehydration, and unintended weight loss must be considered. Finally, long‐term accessibility and cost of incretin‐based therapies represent practical barriers for chronic neurodegenerative indications.

## CONFLICTS OF INTEREST STATEMENT

The authors declare no conflicts of interest.

## Data Availability

As this is a review article, there is no experimental data.
